# Tyrosine kinase inhibitor use and risk of neurodegenerative disease: A Swedish population‐based cohort study

**DOI:** 10.1002/alz.71916

**Published:** 2026-10-11

**Authors:** Felix Wittström, Enoch Yi‐Tung Chen, Iris Blotenberg, Torsten Dahlén

**Affiliations:** ^1^ Centre For Pharmacoepidemiology Department of Medicine Solna Karolinska Institutet Stockholm Sweden; ^2^ Division of Clinical Epidemiology Department of Medicine Solna Karolinska Institutet Stockholm Sweden; ^3^ Department of Medical Epidemiology and Biostatistics Karolinska Institutet Stockholm Sweden; ^4^ German Center for Neurodegenerative Diseases (DZNE) Greifswald Germany; ^5^ Department of Hematology Karolinska University Hospital Solna Stockholm Sweden

**Keywords:** chronic myeloid leukemia, cohort study, neurodegenerative disease, pharmacoepidemiology, tyrosine kinase inhibitors

## Abstract

**INTRODUCTION:**

Effective disease‐modifying therapies remain unavailable for neurodegenerative diseases (NDDs). Tyrosine kinase inhibitors (TKIs) indicated for chronic myeloid leukemia (CML) have been hypothesized to modulate the pathological mechanisms underlying neurodegeneration. Therefore, we aimed to explore the association between initiation of six different TKIs and the subsequent risk of developing NDDs in Swedish nationwide registries (2005–2024).

**METHODS:**

CML patients ≥50 years at diagnosis initiating TKI therapy were identified, alongside five comparators without CML matched on sex, age, and residence at diagnosis. CML cases and comparators were followed from the initiation date of the respective TKI and could contribute follow‐up time to multiple TKI cohorts. A composite measure of NDD diagnosis or death due to NDD was the outcome. Hazard ratios (HRs) were estimated using Cox proportional hazards models, with adjustments for calendar year at follow‐up initiation, and relevant comorbidities.

**RESULTS:**

A total of 1322 CML cases were included in the study. 1188 initiated imatinib (of which 41 experienced an NDD event), 541 dasatinib (15 events), 390 nilotinib (five events), 174 bosutinib, 71 ponatinib, and 41 asciminib. Adjusted hazard ratios comparing each TKI group to their matched comparator populations were 0.98 (95% confidence interval [CI], 0.71 to 1.36) for imatinib users, 1.18 (95% CI, 0.68 to 2.03) for dasatinib users, and 0.37 (95% CI, 0.15 to 0.90) for nilotinib users. Bosutinib, ponatinib, and asciminib users were too few to conduct comparative analyses.

**DISCUSSION:**

Initiation of nilotinib, but not imatinib or dasatinib, was associated with a significantly lower NDD incidence relative to general population comparators.

## Highlights

- Tyrosine kinase inhibitors for chronic myeloid leukemia may affect neurodegeneration.
- The pre‐clinical evidence is compelling, but studies with long follow up are needed.
- We followed 1322 chronic myeloid leukemia patients and general population matched comparators.
- Nilotinib use was associated with lower incidence of neurodegenerative disease.
- Use of imatinib or dasatinib was not, and users of other drugs were too few to study.

## 1 BACKGROUND

As populations around the world age, the number of individuals affected by neurodegenerative diseases (NDDs)—most prominently Alzheimer's disease and Parkinson's disease—continues to rise steadily.^1^, ^2^ Although several treatment options exist that alleviate symptoms, the need for effective disease‐modifying treatments remains substantial.^3^, ^4^, ^5^

Breakpoint cluster region‐Abelson 1 tyrosine kinase inhibitors (BCR::ABL1 TKIs), hereafter TKIs, are primarily used in chronic myeloid leukemia (CML),^6^ but may also affect neurodegenerative processes such as clearance of neurotoxic proteins.^7^

TKIs vary in their tyrosine kinase target selectivity and ability to cross the blood–brain barrier.^7^ Imatinib, the first‐generation TKI widely used in CML, does not cross the blood–brain barrier,^8^ and laboratory data suggest that it lacks disease‐modifying activity in Alzheimer's disease.^9^ In contrast, newer‐generation TKIs (dasatinib, nilotinib, bosutinib, ponatinib, and asciminib) cross the blood–brain barrier and have been shown to have a positive effect on biomarkers in Alzheimer's and Parkinson's diseases.^7^, ^10^, ^11^, ^12^, ^13^

Taken together, previous studies suggest that specific TKIs may influence pathological processes underlying NDDs. However, clinical trial data are limited, reflecting the large cohorts and long‐follow up periods needed to investigate an effect on NDD incidence^3^, ^4^. Furthermore, early intervention is likely required, as the underlying neurodegenerative processes begin years before symptoms arise^3^, ^4^. This temporal disconnect limits the ability of trials to capture disease‐modifying effects on disease development and underscores the need for observational studies.

To address this knowledge gap, we conducted a population‐based cohort study using the Swedish CML Register, which provides nearly complete nationwide coverage of CML patients and a follow‐up period of up to 19 years. This study aimed to explore the association between initiation of six different TKIs and the subsequent risk of developing NDDs. To do so, we compared the risk of NDD in CML patients initiating treatment with imatinib, dasatinib, nilotinib, bosutinib, ponatinib, and asciminib relative to general population comparators.

## 2 METHODS

### 2.1 Setting, design, and data sources

This study used a matched cohort design. We identified patients with a diagnosis of CML July 1, 2005, to December 31, 2024, in the Swedish CML Register. For every patient, five individuals without CML were matched by sex, as well as age and municipality of residence at diagnosis, at random from the Swedish Total Population Register. Given the negligible risk of NDD in younger ages, we limited the study population to those with a diagnosis ≥ 50 years of age^3^, ^4^. Given the research question, we excluded CML patients without TKI treatment. Cases and comparators could contribute follow‐up time to multiple TKI cohorts, depending on the TKI use of the cases over time. Cases and comparators with a history of NDD prior to the start of follow‐up (date of TKI initiation) were excluded. A study flowchart is depicted in Figure 1. Using the civil personal registration number, assigned to each resident at birth or upon immigration,^14^ we linked data from the Swedish Prescribed Drug Register (PDR), Swedish National Patient Register (NPR) and the Swedish Cause of Death Register (CDR).^15^, ^16^, ^17^, ^18^

**RESEARCH IN CONTEXT**

1. **Systematic review**: The authors reviewed the literature using PubMed. Tyrosine kinase inhibitors targeting BCR::ABL1, have been proposed to affect neurodegenerative processes, with compelling pre‐clinical evidence, but divergent findings in recently published trials.
2. **Interpretation**: In this 19‐year nationwide Swedish cohort study, initiation of nilotinib for chronic myeloid leukemia, but not imatinib or dasatinib, was associated with a significantly lower incidence of neurodegenerative disease relative to general population comparators. Our findings provide the largest, long‐term clinical evidence to date for tyrosine kinase inhibitors’ repurposing potential in neurodegenerative disease prevention. A limitation of the study is differential health‐care usage and diagnostic investigations of patients using tyrosine kinase inhibitors as compared to general population comparators.
3. **Future directions**: Our study provides important evidence to support further trials of nilotinib in high‐risk populations of neurodegenerative disease, with careful consideration of intervention timing and dosing, to prevent disease development.

**FIGURE 1 alz71916-fig-0001:**
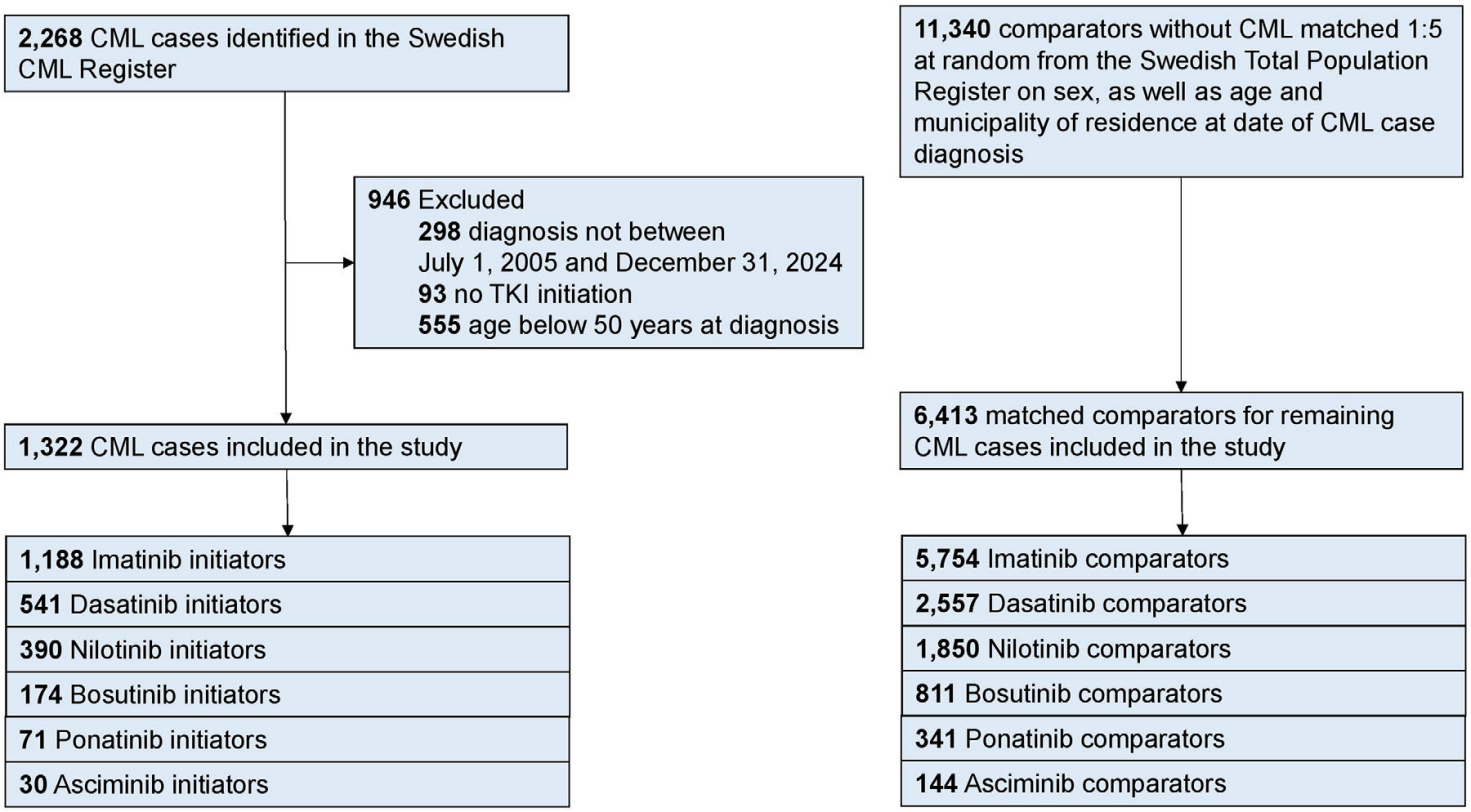
Study flowchart.

The Swedish CML Register covers approximately 98% of CML cases since 2002 and includes information on age, sex, residence and date of diagnosis.^17^ The PDR holds data on dispensed prescriptions since July 1, 2005, coded according to the *Anatomical Therapeutic Chemical* (ATC) system.^16^ The NPR contains diagnoses given in in‐patient care and out‐patient hospital‐based specialist care, coded according to the International Classification of Diseases (ICD).^18^ The CDR includes data on deaths in Sweden, with causes of death registered with ICD‐codes.^15^

### 2.2 Exposure

Six exposure categories of TKIs were considered based on prescription dispensations in the PDR: imatinib, dasatinib, nilotinib, bosutinib, ponatinib, and asciminib.

In CML, switching of TKI treatment is common. Imatinib is generally the first‐line treatment, with some patients switching to or between newer‐generation TKIs, due to intolerable side effects or resistance to treatment.^19^, ^20^ Patients were considered as users of imatinib, dasatinib, nilotinib, bosutinib, ponatinib, or asciminib starting from the date they first filled a dispensation of that TKI until the date they filled a dispensation for another TKI (i.e., switched treatment). Due to the stepwise nature of CML treatment, limiting inclusion only to first‐line treatments may introduce selection bias. That is, patients using newer‐generation TKIs as a first‐line treatment may differ systematically from those who use these drugs in later lines of treatment.^21^ To minimize bias, CML patients contributed follow‐up time to multiple exposure categories based on their sequential TKI use.

### 2.3 Outcome

The primary outcome was a composite measure of NDD diagnosis or death due to NDD. Information regarding NDD diagnosis came from the NPR (with diagnostic codes in either main or secondary position, as an inpatient or outpatient) and death due to NDD from the CDR (with diagnostic codes in either main, secondary, or tertiary position). The composite outcome was chosen to increase statistical efficiency.^22^

To address potential misclassification between dementia and parkinsonian disorders, we included all diagnostic codes indicative of these conditions as our primary outcome.^23^, ^24^ Codes indicating vascular dementia were excluded from this definition, due to the distinct non‐neurodegenerative pathology behind vascular dementia, as well as the increased risk of vascular events associated with certain TKI treatments.^25^ In two Swedish validation studies, diagnostic codes for all‐cause dementia had a positive predictive value (PPV) of 81.3% and 99.0% in the NPR and CDR, respectively, and diagnostic codes for all‐cause parkinsonian disorder had a PPV of 88.0% and 94.4%.^23^, ^24^, [Fig alz71916-fig-0001]

### 2.4 Covariates

To adjust for confounding in our analyses, we used information on comorbidities that may affect the risk of NDDs.^3^, ^4^, ^5^ This included type 1 or 2 diabetes mellitus, hypertension, obesity, hearing loss, alcohol use disorder, malignant melanoma, and chronic obstructive pulmonary disease codes registered in the NPR at any point prior to start of follow‐up.

### 2.5 Main analysis

Baseline characteristics of the cases in the six TKI exposure categories and their matched comparators were described. Incidence rates of NDD were compared between each TKI exposure and their respective matched comparators. Follow‐up began at the date of initiation of the respective TKI for CML patients, and at the corresponding date for matched comparators. Cases and comparators were followed until incident NDD event, death, emigration, or December 31, 2024, whichever came first. Additionally, TKI users were censored upon initiation of any other TKI in an ‘as‐treated’ follow‐up approach.

For imatinib, dasatinib, and nilotinib, we estimated unadjusted (crude) cumulative incidence functions using the Aalen–Johansen method, accounting for the competing risk of death, and calculated the cumulative risk of NDD at attained age 95 years.^22^ For bosutinib, ponatinib, and asciminib, only descriptive characteristics, total follow‐up time, and number of events were reported, due to the low number of users.

We used the Cox proportional hazards model to estimate hazard ratios (HRs) with associated 95% confidence intervals (CIs), comparing patients with imatinib, dasatinib, and nilotinib use to their respective comparator populations. The models were constructed with age as the underlying time scale, as age is a primary determinant of NDD risk. Analyses were performed crude comparing the matched populations, as well as additionally adjusted for sex, year at start of follow‐up, age at start of follow‐up, as well as comorbidities that may affect the risk of NDDs. Sex and age were included as covariates in the adjusted models to account for changes in group composition between CML diagnosis (time of matching) and TKI initiation (start of follow‐up).^26^ We assessed the proportional hazards assumption using scaled Schoenfeld residuals. In addition to the Grambsch–Therneau test, reported globally and for each covariate, we plotted the scaled Schoenfeld residuals for the exposure against attained age, with a smoothed estimate of the time‐varying coefficient and its 95% confidence band, and examined whether this deviated from the constant log‐hazard ratio.

### 2.6 Sensitivity analyses

We performed four sensitivity analyses. First, to explore the impact of differential switching between different TKIs, we changed the “as‐treated” follow‐up approach in the main analysis to an “as‐started” follow‐up approach^27^, where TKI users were not censored at the initiation of any other TKI.

Second, to address reverse causation, where early neurodegenerative symptoms may impact TKI treatment choices, as well as to explore treatment duration effects, we introduced a lag time for the exposure of (i) 6 months and (ii) 12 months.^28^ Follow‐up began 6/12 months after initiation of the TKI for cases, and the corresponding date for the matched comparators.

Third, to address misclassification of the outcome, we used two alternative outcome definitions: (i) We analyzed the outcome defined as ICD codes specifically for Alzheimer's and Parkinson's diseases. These codes show a PPV of 56.6% and 65.7% for Alzheimer's and 70.8% for Parkinson's disease.^23^, ^24^ (ii) We analyzed the outcome defined as medication use indicative of Alzheimer's and Parkinson's disease, as this may capture cases in primary care, where much of dementia care is conducted. A validation study from Finland showed a PPV of 97.1% for drug dispensations indicating Alzheimer's disease.^29^

Fourth, to rule out potential confounding due to frailty we performed a negative control outcome analysis.^30^ As patients with early impairments in cognitive functioning may be more frail, they may receive different CML treatment than their less frail counterparts, and such an association may influence the incidence of neurodegenerative events in the three exposure categories. For this negative control outcome, we analyzed the outcome decubitus ulcer.^31^ Codes used to define exposure, outcomes and covariates can be found in Table S1.

Additionally, we conducted a post hoc active comparator analysis, addressing potential inherent differences between CML patients and the general population. For this analysis we compared outcome incidence rates across person‐time under treatment with different TKIs within the CML cohort. This analysis is not subject to the biases of a user vs. non‐user comparison, but may still be subject to confounding by indication between TKIs.

## 3 RESULTS

A total of 1322 CML cases were included in the study: 1188 initiated imatinib, 541 dasatinib, 390 nilotinib, 174 bosutinib, 71 ponatinib, and 41 asciminib during the study period (Table 1a and 1b). Cases in the six exposure categories were overall similar to each other in terms of proportion of females as well as age at start of follow‐up, with the exception of ponatinib users being younger. As expected, year of CML diagnosis varied markedly between the different TKI user exposure categories according to year of approval in Sweden. TKI users had an overall higher prevalence of diabetes mellitus, hypertension, hearing loss, and chronic obstructive pulmonary disease compared to their matched comparators. The prevalence of obesity and alcohol use disorder was similar in cases and comparators.

**TABLE 1a:** Characteristics of CML cases with imatinib, dasatinib, and nilotinib use and matched comparators from the general population.

Characteristics	Imatinib users	Imatinib comparators	Dasatinib users	Dasatinib comparators	Nilotinib users	Nilotinib comparators
Total individuals, n	1188	5754	541	2557	390	1850
Female* (%)	539 (45.4)	2608 (45.3)	258 (47.7)	1219 (47.7)	187 (47.9)	893 (48.3)
Mean age at start of follow‐up*, years (SD)	68.5 (10.2)	67.95 (10.1)	66.7 (9.8)	66.2 (9.6)	66.7 (9.7)	66.2 (9.5)
**Year of CML diagnosis (%)**						
2005‐2010	294 (24.7)		118 (14.8)		39 (10.0)	
2011‐2015	313 (26.3)		192 (24.0)		180 (46.2)	
2016‐2020	337 (28.4)		267 (33.4)		113 (29.0)	
2021‐2024	244 (20.5)		223 (27.9)		58 (14.9)	
**Comorbidity (%)**						
Diabetes mellitus	174 (14.6)	521 (9.1)	69 (12.8)	219 (8.6)	35 (9.0)	170 (9.2)
Hypertension	450 (37.9)	1400 (24.3)	201 (37.2)	612 (23.9)	138 (35.4)	460 (24.9)
Obesity	59 (5.0)	153 (2.7)	18 (3.3)	94 (3.7)	13 (3.3)	56 (3.0)
Hearing loss	111 (9.3)	429 (7.5)	61 (11.3)	205 (8.0)	29 (7.4)	129 (7.0)
Alcohol use disorder	34 (2.9)	160 (2.8)	15 (2.8)	88 (3.4)	8 (2.1)	55 (3.0)
Malignant melanoma	17 (1.4)	48 (0.8)	12 (2.2)	19 (0.7)	4 (1.0)	14 (0.8)
COPD	60 (5.1)	187 (3.2)	28 (5.2)	75 (2.9)	24 (6.2)	67 (3.6)

Abbreviations: CML, chronic myeloid leukemia; COPD, chronic obstructive pulmonary disorder; SD, standard deviation.

* CML cases and comparators were matched on sex, as well as age and municipality of residence at CML diagnosis.

**TABLE 1b:** Characteristics of CML cases with bosutinib, ponatinib, and asciminib use and matched comparators from the general population.

Characteristics	Bosutinib users	Bosutinib comparators	Ponatinib users	Ponatinib comparators	Asciminib users	Asciminib comparators
Total individuals, *n*	174	811	71	341	30	144
Female* (%)	82 (47.1)	385 (47.5)	35 (49.3)	164 (48.1)	13 (43.3)	62 (43.1)
Mean age at start of follow‐up*, years (SD)	69 (9.6)	68.8 (9.3)	61.1 (8.5)	61.1 (8.3)	64.75 (9.5)	64.75 (9.4)
**Year of CML diagnosis (%)**						
2005–2010	0 (0.0)		0 (0.0)		0 (0.0)	
2011–2015	22 (12.6)		8 (11.3)		0 (0.0)	
2016–2020	67 (38.5)		28 (39.4)		0 (0.0)	
2021–2024	85 (48.9)		35 (49.3)		30 (100.0)	
**Comorbidity (%)**						
Diabetes mellitus	33 (19.0)	79 (9.7)	5 (7.0)	24 (7.0)	2 (6.7)	10 (6.9)
Hypertension	84 (48.3)	235 (29.0)	20 (28.2)	64 (18.8)	16 (53.3)	34 (23.6)
Obesity	14 (8.0)	31 (3.8)	0 (0.0)	15 (4.4)	1 (3.3)	10 (6.9)
Hearing loss	26 (14.9)	88 (10.9)	11 (15.5)	31 (9.1)	2 (6.7)	16 (11.1)
Alcohol use disorder	5 (2.9)	29 (3.6)	1 (1.4)	14 (4.1)	0 (0.0)	5 (2.9)
Malignant melanoma	7 (4.0)	10 (1.2)	2 (2.8)	1 (0.3)	2 (6.7)	7 (4.0)
COPD	10 (5.7)	34 (4.2)	2 (2.8)	9 (2.6)	0 (0.0)	10 (5.7)

Abbreviations: CML, chronic myeloid leukemia; COPD, chronic obstructive pulmonary disorder; SD, standard deviation.

* CML cases and comparators were matched on sex, as well as age and municipality of residence at CML diagnosis.

### 3.1 Main Analysis

We identified 41, 15, and five NDD events among imatinib, dasatinib, and nilotinib users, while there were no events among bosutinib, ponatinib, or asciminib users. This corresponded to incidence rates per 1000 person‐years of 8.3 (95% CI, 6.0 to 11.3) for imatinib, 8.2 (95% CI, 4.6 to 13.5) for dasatinib, and 2.9 (95% CI, 0.9 to 6.8) for nilotinib (Table 2). Figure 2 presents cumulative incidence functions for the three cohorts while treating death as a competing event. At attained age 95 years, the cumulative risk of NDD was 15.3% (95% CI, 11.5% to 20.4%) for imatinib users, 14.4% (95% CI, 8.9% to 23.4%) for dasatinib users, and 5.7% (95% CI, 2.4% to 13.4%) for nilotinib users. For the respective matched comparators, the cumulative risk of NDD was 22.1% (95% CI, 20.1% to 24.3%), 24.7% (95% CI, 20.7% to 29.3%), and 24.5% (95% CI, 20.0% to 30.0%), respectively. The adjusted HRs comparing each TKI group to their matched comparator populations were 0.98 (95% CI, 0.71 to 1.36) for imatinib users, 1.18 (95% CI, 0.68 to 2.03) for dasatinib users, and 0.37 (95% CI, 0.15 to 0.90) for nilotinib users (Table 2). There was no evidence against the proportional hazards assumption in any of the models, either globally or for individual covariates.

**TABLE 2:** Risk of a composite measure of NDD in CML cases with imatinib, dasatinib, nilotinib, bosutinib, ponatinib, and asciminib use, and their matched comparators from the general population.

Cohort^*^	Follow‐up, person‐years	Events, No.	Incidence rate per 1000 person‐years (95% CI)	Crude HR^*^ (95% CI)	Adjusted HR (95% CI)
Imatinib					
CML cases	4933	41	8.3 (6.0 to 11.3)	1.03 (0.74 to 1.42)	0.98 (0.71 to 1.36)
Comparators	44,466	379	8.5 (7.7 to 9.4)	Reference	Reference
Dasatinib					
CML cases	1829	15	8.2 (4.6 to 13.5)	1.22 (0.71 to 2.09)	1.18 (0.68 to 2.03)
Comparators	16315	116	7.1 (5.9 to 8.5)	Reference	Reference
Nilotinib					
CML cases	1704	5	2.9 (0.9 to 6.8)	0.39 (0.16 to 0.97)	0.37 (0.15 to 0.90)
Comparators	14,372	104	7.2 (5.9 to 8.8)	Reference	Reference
Bosutinib					
CML cases	444	0	0.0 (0.0 to 8.3)	—	—
Comparators	3326	18	5.4 (3.2 to 8.6)	—	—
Ponatinib					
CML cases	201	0	0.0 (0.0 to 18.4)	—	—
Comparators	1403	4	2.8 (0.8 to 7.3)	—	—
Asciminib					
CML cases	25	0	0.0 (0.0 to 147.6)	—	—
Comparators	138	0	0.0 (0.0 to 26.7)	—	—

*Note*: Adjusted Model: adjusted by sex, age at TKI initiation, calendar year of TKI initiation, and comorbidities, including diabetes, hypertension, obesity, hearing loss, malignant melanoma, and COPD.

* CML cases and comparators were matched on sex, as well as age and municipality of residence at CML diagnosis.

Abbreviations: CI, confidence intervals; CML, chronic myeloid leukemia; COPD, chronic obstructive pulmonary disease; HR, hazard ratio; NDD, neurodegenerative disease.

**FIGURE 2:**
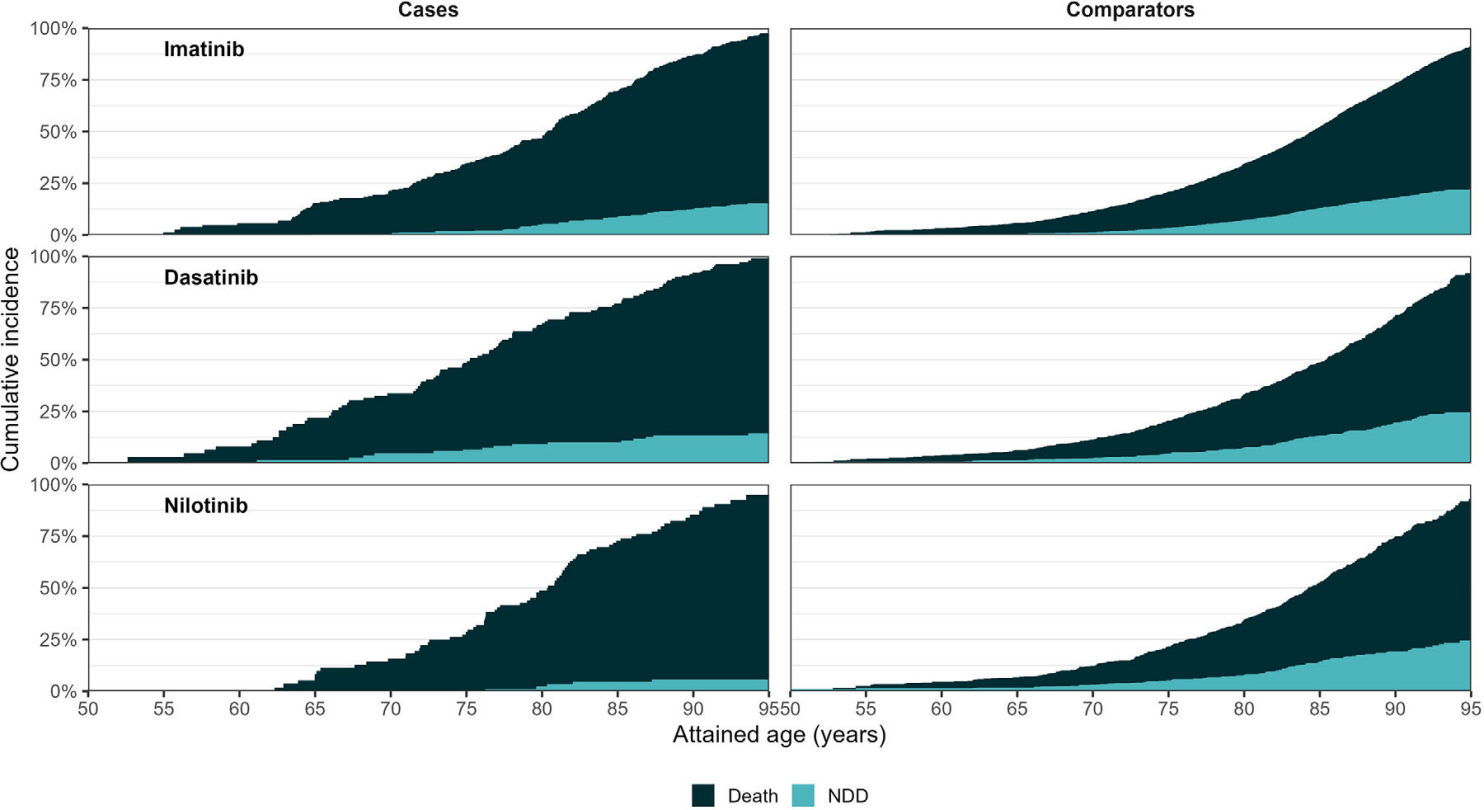
Crude Aalen–Johansen cumulative incidence functions of neurodegenerative disease, with death as a competing event. Separate curves for patients with chronic myeloid leukemia with imatinib, dasatinib, and nilotinib use; and their corresponding comparators matched on sex, as well as age and municipality of residence at diagnosis.

### 3.2 Sensitivity analyses

In the “as‐started” analyses results were similar to the main analysis, with adjusted HRs of 0.90 (95% CI, 0.68 to 1.18) for imatinib users, 0.88 (95% CI, 0.53 to 1.48) for dasatinib users, and 0.36 (95% CI, 0.17 to 0.78) for nilotinib users (Table S2, analysis 1). In the lag time analyses, incidence rates for all TKI users increased stepwise, while remaining stable for comparators. Incidence rates for imatinib users were 8.9 (95% CI, 6.3 to 12.1) with a 6‐month lag time and 9.5 (95% CI, 6.7 to 13.1) with a 12‐month lag time, for dasatinib users 8.8 (95% CI, 4.8 to 14.8) and 10.0 (95% CI, 5.5 to 16.9), for nilotinib users 3.3 (95% CI, 1.1 to 7.6) and 3.6 (95% CI, 1.2 to 8.5).‐ (Table S2, analysis 2a and 2b).

Using other outcome definitions changed the estimates, while the overall direction of the results remained similar to the main analysis. (i) In the analyses defining the outcome with ICD codes specific for Alzheimer's and Parkinson's disease, we identified 15, five, and two events among imatinib, dasatinib, and nilotinib users, respectively. The corresponding adjusted HRs were 0.68 (95% CI, 0.40 to 1.16) for imatinib users, 0.78 (95% CI, 0.31 to 1.97) for dasatinib users, and 0.34 (95% CI, 0.08 to 1.41) for nilotinib users (Table S2, analysis 3a). (ii) When defining the outcome based on medication use indicative of Alzheimer's and Parkinson's disease, we identified 21, nine, and two events among imatinib, dasatinib, and nilotinib users, respectively. The corresponding adjusted HRs were 0.70 (95% CI, 0.45 to 1.09) for imatinib users, 0.99 (95% CI, 0.50 to 1.98) for dasatinib users, and 0.21 (95% CI, 0.05 to 0.84) for nilotinib users (Table S2, analysis 3b).

In the negative control outcome analysis, we observed 17, six, and six decubitus ulcer events among imatinib, dasatinib, and nilotinib users, respectively. Adjusted HRs were 1.90 (95% CI, 1.12 to 3.23) for imatinib users, 2.28 (95% CI, 0.91 to 5.71) for dasatinib users, and 2.02 (95% CI, 0.81 to 5.03) for nilotinib users compared to their respective comparators (Table S2, analysis 4).

In the *post hoc* analysis, the crude incidence rate ratio (IRR) of NDD comparing dasatinib users to imatinib users was 0.99 (95% CI, 0.55 to 1.78) and comparing nilotinib users to imatinib users was 0.35 (95% CI, 0.14 to 0.89). The IRRs of the two alternative NDD outcome definitions showed similar direction of the estimates while the intervals included unity. The IRRs of decubitus ulcer were 0.97 (95% CI, 0.38 to 2.45) for dasatinib vs imatinib and 1.04 (95% CI, 0.41 to 2.64) for nilotinib vs. imatinib, Table S3.

## 4 DISCUSSION

In this Swedish population‐based cohort study of CML patients, nilotinib initiation was associated with a significantly lower incidence of a composite measure of NDD compared to matched general population comparators. These findings remained robust across multiple sensitivity analyses, which addressed differential treatment switching, reverse causality, and outcome misclassification. In contrast, imatinib and dasatinib initiation showed no substantial association with NDD incidence relative to their matched comparators. Comparative analyses for bosutinib, ponatinib, and asciminib users were precluded by insufficient cohort sizes.

These results are consistent with a nationwide cohort study from South Korea, which utilized insurance claim data to compare 5579 TKI users with 27,895 never‐users. That study reported a 35% reduction in dementia risk among TKI users (adjusted competing risk hazard ratio 0.65, [95% CI, 0.48 to 0.88]).^32^ However, unlike our findings, the South Korean study observed similar adjusted hazard ratios for all TKIs examined (imatinib, nilotinib, radotinib, dasatinib). This discrepancy may be attributable to differences in study design, particularly the lack of adjustment for treatment switching, which could allow the potential effect on neurodegenerative processes of one TKI to influence the observed associations for other TKIs. Both studies are limited by the comparison to non‐users; however, our results for specific TKIs are strengthened by the *post hoc* active comparator analysis, showing a significantly lower crude IRR in CML patients during treatment with nilotinib versus treatment with imatinib, while the IRR for the negative control outcome was near null. The lag time analyses highlight potential differences in health‐care usage and diagnostic investigations of CML patients as compared to general population comparators.

The observed cumulative risk of NDD while treating death as a competing event indicate the relevance of our findings in a clinical setting. In a survey study of dementia risk from 55 years of age onward with mortality treated as a competing event, 42% (95% CI, 41% to 43%) developed dementia by 95 years.^33^ This is in line with the risk observed in the matched comparator populations at around 23%, given that in the survey study approximately half of the events were identified in hospital or death records, and the other half was identified in interviews.

Based on pre‐clinical data mechanisms behind the findings may be that TKIs affect neurotoxic protein clearance and regulate the neuronal immune response.^7^ Imatinib does not penetrate the blood–brain barrier, and a clinical trial in humans did not show an effect on plasma levels of amyloid beta, a biomarker of neurodegeneration^8^, ^9^. Dasatinib penetrates the blood–brain barrier^34^, but may primarily affect neurodegenerative processes through targeting senescent cells.^35^ It has been investigated for NDD prevention in combination with other senolytic compounds such as *quercetin*, which is not used in CML treatment.^11^ Nilotinib has limited penetrance of the blood–brain barrier, but even at low doses prevents neuronal loss in pre‐clinical models of NDDs.^7^ Furthermore, nilotinib has been investigated in several clinical trials, showing an effect on multiple biomarkers of neurodegeneration such as increased cerebrospinal fluid levels of dopamine metabolites (homovanillic acid and 3,4‐dihydroxyphenylacetic acid), and reductions in α‐synuclein, amyloid beta, and hyperphosphorylated tau.^12^, ^36^, ^37^ In a meta‐analysis of five trials (*n* = 197) nilotinib showed an effect size of −1.15 (95% CI, −3.00 to 0.69) on Parkinson's disease and Lewy body dementia symptom progression at a daily dose of 300 mg, unlike lower doses and other TKIs.^38^ Notably, this dose is below the daily dose used in CML (often 600 mg daily).

Further evidence of the effect of TKIs on neurodegeneration comes from a case report of three CML patients with mixed prior TKI use, but notably all with prior nilotinib use, which developed symptoms of cognitive dysfunction after TKI discontinuation.^39^ This may potentially reflect previously suppressed neurodegenerative processes resuming upon stopping TKI use. While the findings of this case report are limited to three patients, it further strengthens the hypothesis that TKIs may affect neurodegeneration.

A review of current evidence on the role of protein kinases in NDDs highlights the complexity of drug development in this area.^40^ Factors such as kinase target selectivity, dosing strategy, blood–brain barrier penetration, and the timing of the intervention window may contribute to conflicting results from trials, including studies of nilotinib, which have shown mixed results.^38^

### 4.1 Strengths and limitations

A strength of our study is the use of a population‐based cancer register with high coverage. This lowered the risk of bias resulting from factors affecting selection into the cohort, such as access to healthcare.^41^ However, despite the cohort size and long follow up the outcome was rare, limiting our comparative analyses only to users of imatinib, dasatinib, and nilotinib.

Another strength is the use of multiple outcome definitions. The consistent direction of estimates across both broader and more specific diagnostic definitions, as well as definition through medication use strengthens our findings. However, NDD is difficult to capture accurately using register data: there may be a long lag time between symptom onset and registration of the diagnostic code, with misclassification between the diagnostic codes further contributing to lower validity.^23^, ^24^ This limited our ability to investigate specific NDDs, and our results should be interpreted in general for the NDD composite measure and not specifically Alzheimer's or Parkinson's disease.

Modifiable risk factors such as smoking, alcohol use, diet, or physical activity may also influence the risk of NDD^42^. Our study lacked information about these variables, which may contribute to residual confounding. However, the adjustment for related comorbidities most likely minimized this source of bias.

Another limitation is the comparison between CML patients and the general population. This may bias the results in two directions, making the diagnosis of NDD more common in cases (increased healthcare contact), or make the outcome less common in cases (clinicians may avoid investigating cognitive decline in a patient with leukemia and limited life expectancy). Increased healthcare contact is potentially reflected in the increased risk of decubitus ulcer observed for imatinib, dasatinib and nilotinib users. This bias should, however, be equal for the different TKIs, as reflected in the active comparator analyses yielding IRRs near null for the negative control outcome. When considering the lag time analyses and comparing the cumulative risks of NDD at 95 years in all three TKI groups (15.3% for imatinib, 14.4% for dasatinib, and 5.7% for nilotinib) to the risks for the matched comparators (between 22% and 25%), there is some indication that diagnostic bias may affect the results. While such a bias means the absolute risks should be interpreted with caution, it should again be equal between TKI groups and, therefore, does not explain away our findings of a signal of reduced NDD incidence among nilotinib initiators, which was not present for imatinib or dasatinib initiators, as highlighted by our active comparator analysis. An analysis of accounting for treatment duration or cumulative dose would be helpful to elucidate drug specific effects but was precluded due to the cohort size.

### 4.2 Conclusions

These findings indicate that in patients with CML, initiation of nilotinib, but not imatinib or dasatinib, was associated with a significantly lower incidence of NDDs relative to general population comparators. Despite compelling pre‐clinical evidence, several trials of TKIs have failed to demonstrate significant effects on NDD symptom progression. Therefore, this study provides important evidence to support further trials of nilotinib in high‐risk populations, with careful consideration of intervention timing and dosing, to prevent disease development.

## AUTHOR CONTRIBUTIONS

Wittström, Chen, and Dahlén were responsible for the conception of the study. Wittström, Chen, Blotenberg, and Dahlén developed the methodology used. Dahlén takes responsibility for the data acquisition, and Wittström and Chen conducted the data analysis. All authors reviewed and approved the final version for submission. Dahlén is the guarantor.

## CONFLICTS OF INTEREST STATEMENT

Wittström reports participation in research projects funded by Pfizer, all with funds paid to the institution where he is employed and with no relation to the work reported in this paper. Dahlén reports funding unrelated to the current project from Xspray Pharma and Novo Nordisk. The other authors have no conflicts of interest to declare. Author disclosures are available in the Supporting Information.

## ETHICAL APPROVAL

The use of registry data for this study was approved by the Swedish Ethical Review Authority (DNR 2021‐00947 and 2024‐02782‐02).

## CONSENT STATEMENT

In Swedish regulation studies utilizing registry data are exempted from the requirement for patient consent.

## Supporting information




**Supporting Information**: alz71916‐sup‐0001‐SuppMat.docx

**Supporting Information**: alz71916‐sup‐0002‐ICJME.pdf
