# Supplementary material for: Tyrosine kinase inhibitor use and risk of neurodegenerative disease: A Swedish population‐based cohort study
Source: Alzheimers Dement. 2026 Oct 11;22(10):e71916. doi: 10.1002/alz.71916 (PMC13655210; doi:10.1002/alz.71916)
Supplement: Supplementary file 2 [file ALZ-22-e71916-s002.docx]

**SUPPLEMENTAL MATERIAL**

**Tyrosine Kinase Inhibitor Use and Risk of Neurodegenerative Disease: A Swedish Population-Based Cohort Study**

**Authors:** Felix Wittström, Enoch Yi-Tung Chen, Iris Blotenberg, Torsten Dahlén

**Table of Contents**

[**Table S1.** International Classification of Diseases (ICD), and Anatomical Therapeutic Chemical Classification System (ATC) codes used in the study.	2](#_Toc232671349)

[**Table S2.** Risk of neurodegenerative disease in 1) main outcome with ‘as-started’ follow-up approach, 2) main outcome with 6 months lag time, 3a) specific Alzheimer’s or Parkinson’s disease outcome, 3b) medication use indicative of Alzheimer’s or Parkinson’s disease outcome as well as 4) a negative control outcome (decubitus ulcer), in CML cases with imatinib, dasatinib and nilotinib use, and their matched comparators.	4](#_Toc232671350)

## **Table S1.** International Classification of Diseases (ICD), and Anatomical Therapeutic Chemical Classification System (ATC) codes used in the study.

|  | **ICD-10** | **ATC** |
| --- | --- | --- |
| **Exposure** |  |  |
| Tyrosine-Kinase Inhibitors |  | L01EA01 Imatinib  L01EA02 Dasatinib  L01EA03 Nilotinib  L01EA04 Bosutinib  L01EA05 Ponatinib  L01EA06 Asciminib |
| **Outcome** |  |  |
| **Main analysis, broad neurodegenerative disease definition** | | |
| Dementia and parkinsonian disorders | F00 Dementia in Alzheimer  F02 Dementia in other diseases  F03 Unspecified dementia  F05.1 Delirium with underlying dementia  G20 Parkinson disease  G21.4 Vascular parkinsonism  G21.8 Other secondary parkinsonism  G21.9 Secondary parkinsonism, unspecified  G23.1 Progressive supranuclear ophthalmoplegia  G23.2 Striatonigral degeneration  G23.9 Unspecified degenerative disease of basal ganglia  G25.9 Unspecified extrapyramidal and movement disorder G30 Alzheimer disease G31.1 Senile degeneration of brain, not elsewhere classified  NOT INCLUDING F01 Vascular dementia |  |
| **Sensitivity analysis, specific neurodegenerative disease definition** | | |
| Alzheimer’s disease or Parkinson’s disease | F00 Dementia in Alzheimer  G20 Parkinson disease  G30 Alzheimer disease |  |
| **Sensitivity analysis, medication use indicative of neurodegenerative disease** | | |
| Alzheimer’s disease or Parkinson’s disease |  | N06D Anti-dementia drugs  N04B Dopaminergic agents *Exluding*: N04BC05 (pramipexol), N04BC09 (rotigotine), N04BA02 (levodopa) due to their use in Restless Legs Syndrome |
| **Negative control outcome analysis** | | |
| Decubitus ulcer | L89 |  |
| **Comorbidities** |  |  |
| Type 1 or 2 diabetes mellitus | E10  E11  E12  E13  E14  G63.2  H36.0  N08.3 |  |
| Hypertension | I10  I11  I12  I13  I14  I15 |  |
| Obesity | E66 |  |
| Hearing loss | H90  H91 |  |
| Alcohol use disorder | F10 |  |
| Malignant melanoma | C43 |  |
| Chronic obstructive pulmonary disease | J41  J42  J43  J44 |  |

## **Table S2.** Risk of neurodegenerative disease in 1) main outcome with ‘as-started’ follow-up approach, 2) main outcome with 6 months lag time, 3a) specific Alzheimer’s or Parkinson’s disease outcome, 3b) medication use indicative of Alzheimer’s or Parkinson’s disease outcome as well as 4) a negative control outcome (decubitus ulcer), in CML cases with imatinib, dasatinib and nilotinib use, and their matched comparators.

| **Sensitivity analysis 1: main outcome with ‘as-started’ follow-up approach** | **Imatinib users** | **Imatinib comparators** | **Dasatinib users** | **Dasatinib comparators** | **Nilotinib users** | **Nilotinib comparators** |
| --- | --- | --- | --- | --- | --- | --- |
| Total follow-up time, years | 8,214 | 44,479 | 2,916 | 16,317 | 2,745 | 14,372 |
| NDD composite measure events, n | 60 | 379 | 17 | 117 | 7 | 104 |
| Incidence rate, per 1000 person-years (95% CI) | 7.3 (5.6, 9.4) | 8.5 (7.7, 9.4) | 5.8 (3.4, 9.3) | 7.2 (5.9, 8.6) | 2.5 (1.0, 5.2) | 7.2 (5.9, 8.8) |
| Crude HR* (95% CI) | 0.92 (0.70, 1.21) | Reference | 0.91 (0.55, 1.52) | Reference | 0.38 (0.18, 0.82) | Reference |
| Adjusted HR (95% CI) | 0.90 (0.68, 1.18) | Reference | 0.88 (0.53, 1.48) | Reference | 0.36 (0.17, 0.78) | Reference |
| **Sensitivity analysis 2: main outcome with 6 months lag time** | **Imatinib users** | **Imatinib comparators** | **Dasatinib users** | **Dasatinib comparators** | **Nilotinib users** | **Nilotinib comparators** |
| Total follow-up time, years | 4,404 | 35,069 | 1,588 | 12,747 | 1,526 | 11,994 |
| NDD composite measure events, n | 39 | 306 | 14 | 95 | 5 | 89 |
| Incidence rate, per 1000 person-years (95% CI) | 8.9 (6.3, 12.1) | 8.7 (7.8, 9.8) | 8.8 (4.8, 14.8) | 7.4 (6.0, 9.1) | 3.3 (1.1, 7.6) | 7.4 (6.0, 9.1) |
| Crude HR* (95% CI) | 1.11 (0.79, 1.54) | Reference | 1.29 (0.74, 2.27) | Reference | 0.46 (0.19, 1.12) | Reference |
| Adjusted HR (95% CI) | 1.05 (0.75, 1.47) | Reference | 1.22 (0.69, 2.16) | Reference | 0.44 (0.18, 1.09) | Reference |
| **Sensitivity analysis 3a:** **specific Alzheimer or Parkinson diagnosis outcome** | **Imatinib users** | **Imatinib comparators** | **Dasatinib users** | **Dasatinib comparators** | **Nilotinib users** | **Nilotinib comparators** |
| Total follow-up time, years | 4,976 | 45,069 | 1,854 | 16,533 | 1,712 | 14,505 |
| Alzheimer or Parkinson disease events, n | 15 | 199 | 5 | 58 | 2 | 50 |
| Incidence rate, per 1000 person-years (95% CI) | 3.0 (1.7, 5.0) | 4.4 (3.8, 5.1) | 2.7 (0.9, 6.3) | 3.5 (2.7, 4.5) | 1.2 (0.1, 4.2) | 3.4 (2.6, 4.5) |
| Crude HR* (95% CI) | 0.71 (0.42, 1.20) | Reference | 0.81 (0.33, 2.03) | Reference | 0.35 (0.08, 1.43) | Reference |
| Adjusted HR (95% CI) | 0.68 (0.40, 1.16) | Reference | 0.78 (0.31, 1.97) | Reference | 0.34 (0.08, 1.41) | Reference |
| **Sensitivity analysis 3b: Medication use indicative of Alzheimer or Parkinson disease outcome** | **Imatinib users** | **Imatinib comparators** | **Dasatinib users** | **Dasatinib comparators** | **Nilotinib users** | **Nilotinib comparators** |
| Total follow-up time, years | 4,975 | 44,736 | 1,823 | 16,396 | 1,703 | 14,338 |
| Prescription dispensations, n | 21 | 271 | 9 | 88 | 2 | 75 |
| Incidence rate, per 1000 person-years (95% CI) | 4.2 (2.6, 6.4) | 6.1 (5.4, 6.8) | 4.9 (2.3, 9.4) | 5.4 (4.3, 6.6) | 1.2 (0.1, 4.2) | 5.2 (4.1, 6.6) |
| Crude HR* (95% CI) | 0.72 (0.46, 1.12) | Reference | 1.00 (0.51, 2.00) | Reference | 0.22 (0.05, 0.90) | Reference |
| Adjusted HR (95% CI) | 0.70 (0.45, 1.09) | Reference | 0.99 (0.50, 1.98) | Reference | 0.21 (0.05, 0.84) | Reference |
| **Sensitivity analysis 4: Negative control outcome of decubitus ulcer diagnosis** | **Imatinib users** | **Imatinib comparators** | **Dasatinib users** | **Dasatinib comparators** | **Nilotinib users** | **Nilotinib comparators** |
| Total follow-up time, years | 5,059 | 46,231 | 1,849 | 16,824 | 1,714 | 14,688 |
| Decubitus ulcer events, n | 17 | 75 | 6 | 25 | 6 | 23 |
| Incidence rate, per 1000 person-years (95% CI) | 3.4 (2.0, 5.4) | 1.6 (1.3, 2.0) | 3.2 (1.2, 7.1) | 1.5 (1.0, 2.2) | 3.5 (1.3, 7.6) | 1.6 (1.0, 2.4) |
| Crude HR* (95% CI) | 2.17 (1.28, 3.68) | Reference | 2.38 (0.98, 5.82) | Reference | 2.23 (0.91, 5.49) | Reference |
| Adjusted HR (95% CI) | 1.90 (1.12, 3.23) | Reference | 2.28 (0.91, 5.71) | Reference | 2.02 (0.81, 5.03) | Reference |
| *Notes:* NDD, Neurodegenerative disease; CML, Chronic myeloid leukemia; CI, confidence intervals; HR, hazard ratio; COPD, chronic obstructive pulmonary disease. Adjusted Model: adjusted by sex, age at TKI initiation, calendar year of TKI initiation, and comorbidities, including diabetes, hypertension, obesity, hearing loss, malignant melanoma, and COPD. *CML cases and comparators were matched on sex, as well as age and municipality of residence at CML diagnosis. | | | | | | |
